# Hemodynamic profile of neonatal fungal sepsis with insights into the disease spectrum: a retrospective study

**DOI:** 10.3389/fped.2026.1905411

**Published:** 2026-08-21

**Authors:** Arshdeep Kaur, Priyadarshini Virupaxi Chougula, Nandini Malshe, Pari Singh, Pradeep Suryawanshi

**Affiliations:** Department of Neonatology, Bharati Vidyapeeth Deemed University Medical College and Hospital, Pune, Maharashtra, India

**Keywords:** candida infection, functional echocardiography, hemodynamics, late-Onset sepsis, neonatal fungal sepsis, preterm neonates, pulmonary hypertension, right ventricular dysfunction

## Abstract

**Background:**

Neonatal fungal sepsis is a significant contributor to late-onset sepsis in neonatal intensive care units, particularly among preterm and low-birth-weight infants. While its epidemiology and microbiology are well described, the hemodynamic profile remains poorly characterized. This study aimed to evaluate the clinical spectrum with a focused assessment of cardiovascular alterations using functional echocardiography.

**Methods:**

Data for this retrospective observational study were collected from a tertiary care neonatal intensive care unit over a three-year period. Neonates with culture-proven fungal sepsis were included. Demographic, clinical, microbiological and outcome data were analyzed. Functional echocardiography was performed during active infection and repeated after microbiological clearance according to unit protocol. Paired hemodynamic comparisons were performed among survivors.

**Results:**

Twenty-six neonates with fungal sepsis were included, of whom 73.1% were preterm and predominantly low birth weight. Non-*Candida albicans* species were the predominant isolates. Respiratory deterioration, feed intolerance and thrombocytopenia were common clinical manifestations, with frequent exposure to prolonged antibiotics, parenteral nutrition and central venous access. Functional echocardiography demonstrated significant cardiovascular involvement during active infection. Survivors showed reduced tricuspid annular plane systolic excursion (6.4 ± 1.2 vs. 7.8 ± 2.4 mm), elevated pulmonary artery systolic pressure (28.5 ± 17.3 vs. 12.8 ± 4.2 mmHg), and increased right ventricular output (315.0 ± 49.4 vs. 246.0 ± 27.1 mL/kg/min), suggesting right ventricular dysfunction with pulmonary vascular involvement and a hyperdynamic circulatory state. Diastolic dysfunction was reflected by lower tricuspid and mitral E/A ratios. Hemodynamic parameters improved following antifungal therapy and microbiological clearance, while left ventricular systolic function remained relatively preserved. Mortality was 19.2%.

**Conclusion:**

In this cohort, neonatal fungal sepsis was associated with reversible hemodynamic alterations, predominantly involving right ventricular function and the pulmonary circulation. Functional echocardiography may provide valuable physiological insights and assist targeted hemodynamic management. Although our study did not include a bacterial sepsis comparator cohort, the observed cardiovascular alterations are broadly consistent with those reported in published neonatal bacterial sepsis studies.

**Clinical trial registration:**

This study was registered with the Clinical Trials Registry of India (CTRI/2025/03/083270) on 23rd march 2025. Trial details are publicly accessible at https://ctri.nic.in/Clinicaltrials/pmaindet2.php?trialid=127824.

## Introduction

Neonatal fungal sepsis, particularly invasive candidiasis, is a leading cause of late-onset sepsis in NICUs and contributes significantly to mortality and long-term morbidity among preterm and low-birth-weight infants (1). Data from the NICHD Neonatal Research Network indicate that invasive candidiasis occurs in approximately 7%–9% of extremely low birth weight (ELBW) infants, underscoring their vulnerability (1, 2). Beyond acute illness, up to 73% of affected ELBW infants experience death or neurodevelopmental impairment, highlighting its lasting impact (1).

The epidemiology of neonatal candidiasis has evolved, with a shift toward non-albicans species. In the multinational NeoOBS cohort from low- and middle-income countries, *Candida albicans* accounted for 35% of isolates, while *C. parapsilosis* (30%) and *C. auris* (14%) were prominent non-albicans species (3). This shift carries therapeutic implications, as non-albicans species are more frequently associated with antifungal resistance, with reports of reduced fluconazole susceptibility among C. *parapsilosis* isolates in LMIC settings (3).

The burden of disease is disproportionately higher in resource-limited settings, where variability in infection control and antimicrobial stewardship contributes to increased incidence. Reported rates range from 0.5%–2% overall but rise to 2%–20% among ELBW infants, with consistently higher estimates in developing countries (4). Neonates in NICUs are exposed to multiple risk factors, including prolonged broad-spectrum antibiotic use, central venous catheters, parenteral nutrition, and invasive respiratory support (1, 2, 5). While often life-saving, these interventions disrupt host defenses and promote fungal colonization, biofilm formation, and invasive dissemination (4). Clinically, neonatal candidiasis presents with nonspecific features overlapping with bacterial sepsis, including respiratory distress, feeding intolerance, apnea, and hemodynamic instability (6, 7).

Despite the central role of cardiovascular dysfunction in neonatal sepsis, the hemodynamic profile of fungal sepsis remains poorly characterized. While functional echocardiographic findings have been described in neonatal bacterial sepsis, comparable data in fungal sepsis are limited, and whether the associated cardiovascular alterations differ between these etiologies remains unclear. Therefore, we aimed to characterize the hemodynamic profile of neonatal fungal sepsis using functional echocardiography and to evaluate changes following microbiological clearance. Given the retrospective single-cohort design, our findings should be considered descriptive, and prospective comparative studies incorporating a bacterial sepsis cohort are needed to determine pathogen-specific hemodynamic differences.

## Methodology

### Study design and setting

This single-center retrospective observational study was conducted over three years (2022–2025) in a tertiary-care NICU in western India. The study was approved by the Institutional Ethics Committee with a waiver of informed consent due to the retrospective design. The study was registered with the Clinical Trials Registry of India (CTRI/2025/03/083270).

### Study population

Neonates (<28 days) with culture-positive fungal sepsis (blood, urine, or cerebrospinal fluid) were included. Diagnosis was defined by the first positive culture. Data were extracted from electronic medical records and microbiology databases using a predefined framework; cases with incomplete records were excluded. As per unit protocol, functional echocardiography is performed at time of clinical suspicion and after treatment of neonatal sepsis; these data were retrieved for analysis. Neonates with primary lung disease or other conditions known to elevate pulmonary artery pressure — such as pneumonia, meconium aspiration syndrome, congenital diaphragmatic hernia, or primary persistent pulmonary hypertension of the newborn — were excluded from the echocardiographic analysis, so that the pulmonary artery systolic pressure measurements reflect the fungal sepsis episode rather than primary respiratory pathology.

### Objectives

The primary objective was to evaluate hemodynamic changes in neonates with fungal sepsis at clinical suspicion (pre) and after culture-confirmed clearance (post). Secondary objectives included assessing clinical, demographic, and laboratory characteristics, risk factors, antifungal sensitivity patterns, and outcomes of affected neonates.

### Sepsis evaluation and microbiology

As per NICU protocol, suspected sepsis was evaluated with a septic screen, blood, urine, and CSF cultures. Samples were processed using the Bact/Alert 3D system (bioMérieux), with species identification and antifungal susceptibility, including MIC determination, performed on the Vitek-2 Compact system using species-specific breakpoints.

### Echocardiographic assessment

Right ventricular function was evaluated using pulmonary artery systolic pressure (PASP), tricuspid annular plane systolic excursion (TAPSE), diastolic function was assessed by tricuspid E/A ratio and global right ventricular performance was measured by using right ventricle output (RVO). Left ventricle systolic function was assessed using fractional shortening (FS), ejection fraction (EF), diastolic function was assessed by mitral E/A ratio and global left ventricular performance was measured by using left ventricle output (LVO). Signs of invasive fungal sepsis were also assessed including valvular/endocarditis changes and repeat echocardiography at culture clearance, as per unit protocol.

### Echocardiographic image acquisition, quality control, and reproducibility

Echocardiographic measurements were performed in accordance with the American Society of Echocardiography (ASE) guidelines for neonatal hemodynamic assessment, using standardized acquisition and analysis protocols (8). Functional echocardiography was performed by echo-trained on duty neonatologists as per unit protocol; more than one operator was therefore involved, and all images were subsequently reviewed by a consultant neonatologist trained in point-of-care ultrasound (POCUS). A pediatric cardiologist performed echocardiography only for endocarditis screening in confirmed cases of invasive fungal infection and did not perform the functional (hemodynamic) assessments. Studies were acquired on a Philips Affiniti 50 ultrasound system (Philips Healthcare) using a 12–4 MHz sector-array transducer. Measurements were obtained from digitally stored images and medical records. Because echocardiography was performed as part of routine unit protocol by the on-duty neonatologist in suspected sepsis cases, blinding of the operator to clinical status was not feasible

### Screening for end-organ involvement

All culture-positive neonates underwent abdominal, renal, and cranial ultrasonography; fundus examination; laboratory evaluation (serum creatinine, electrolytes, bilirubin).

### Data collection

Relevant demographic, clinical, laboratory, microbiological, echocardiographic, and outcome data were extracted from electronic media of medical record department.

### Operational definitions

Fungal sepsis was defined as isolation of fungal organisms from blood, urine, or cerebrospinal fluid culture. Laboratory and hemodynamic parameters were interpreted according to standard neonatal reference ranges. Clinical outcomes, including necrotizing enterocolitis, acute kidney injury, and cholestasis, were defined according to established criteria.

### Statistical analysis

Data were analyzed using IBM SPSS Statistics version 29.0 (IBM Corp., Armonk, NY, USA). Quantitative variables were expressed as mean ± standard deviation (SD) or median with interquartile range (IQR) depending on distribution assessed using the Shapiro–Wilk test. Categorical variables were summarized as frequencies and percentages. Changes in echocardiographic parameters between two groups at the time of clinical suspicion of infection (pre) and after culture clearance (post) were assessed by paired analysis. Normality of the paired differences was assessed using the Shapiro–Wilk test; parameters with normally distributed paired differences were analyzed using the paired t-test, whereas parameters violating the normality assumption were analyzed using the Wilcoxon signed-rank test. Hemodynamic parameters were descriptively reported for survivors and non-survivors due to small sample size. A *p* value <0.05 was considered statistically significant.

## Results

**Figure 1:**
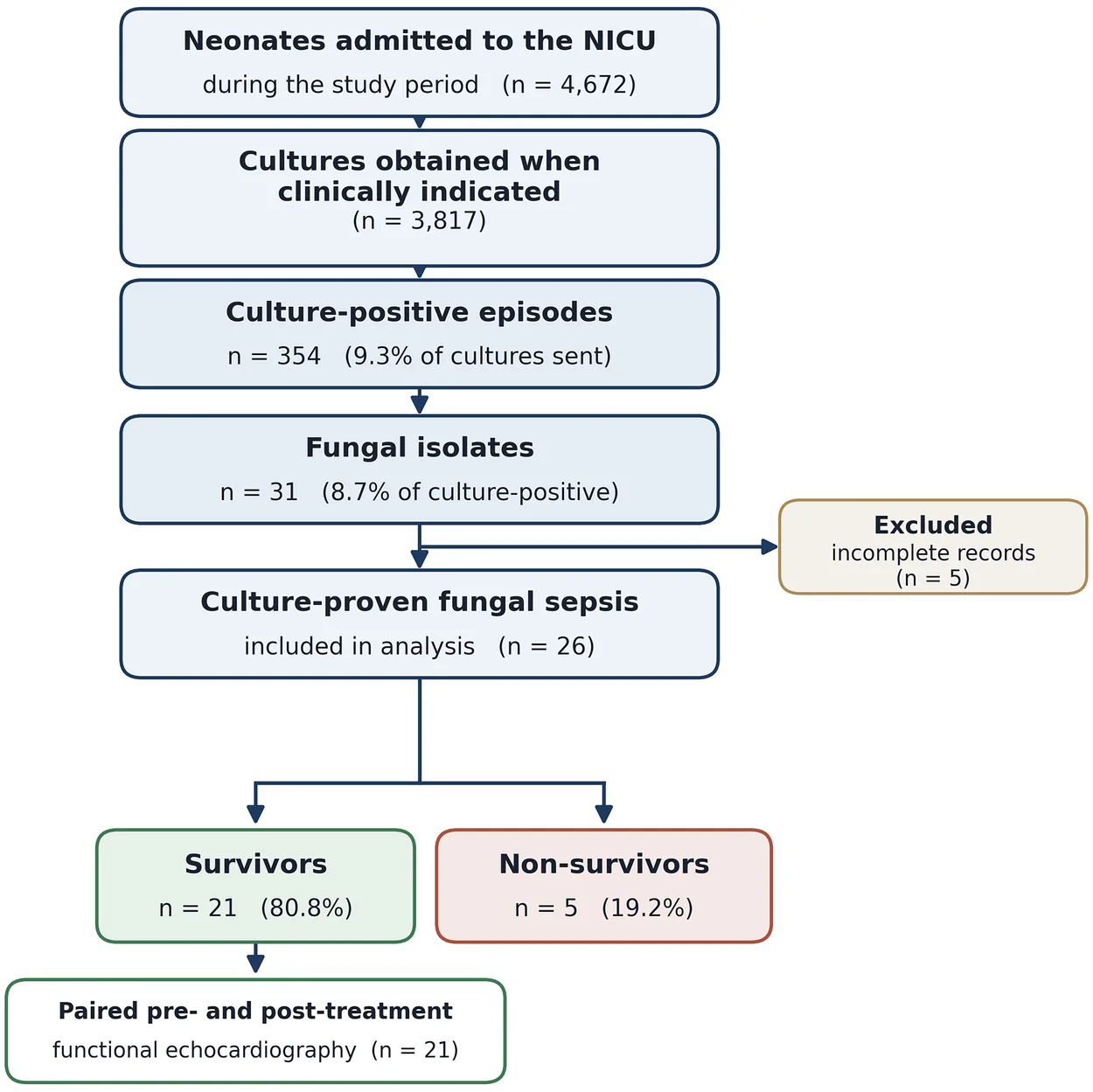
Study flow chart.

During the three-year study period, 4,672 neonates were admitted to the NICU. Blood, urine, cerebrospinal fluid (CSF), and other relevant cultures were obtained when clinically indicated. A total of 3,817 cultures were sent during the study period. Among these, 354 (9.3% of cultures sent) yielded pathogenic organisms, of which 31 (8.7%) were fungal isolates. The 354 culture-positive episodes corresponded to 7.5% of the 4,672 neonates admitted during the study period. Five cases were excluded because of incomplete records, leaving 26 neonates with culture-proven fungal sepsis for the final analysis (Figure 1).

### Baseline characteristics

The study population consisted predominantly of preterm and low-birth-weight infants, with a mean gestational age of 33.3 ± 4.7 weeks and mean birth weight of 1,698 ± 1,017 g. The cohort demonstrated male predominance (76.9%) and was largely composed of outborn infants (84.6%). Most cases presented during the late neonatal period, with a median age at presentation of 24 days (IQR 9–39) (Table 1).

**Table 1:** Baseline demographic, perinatal, and maternal characteristics of the study population.

Variable	Value
Maternal characteristics, *n* (%)	
Primigravida	10 (38.4)
Multigravida	16 (61.5)
Premature rupture of membranes >18 h	3 (11.5)
Gestational diabetes mellitus	2 (7.7)
Pregnancy-induced hypertension	3 (11.5)
Hypothyroidism	1 (3.8)
Gestational age classification, n (%)	
≤28 weeks,	7 (26.9)
29–31 weeks	3 (11.5)
32–33 weeks	4 (15.4)
34–36 weeks	5 (19.2)
≥37 weeks	7 (26.9)
Gestational age (weeks), Mean ± SD	33.3 ± 4.7
Anthropometry and gender, n (%)	
Birth weight (g), Mean ± SD	1,698 ± 1,017
Male	20 (76.9)
AGA	17 (65.4)
SGA	7 (26.9)
LGA	2 (7.7)
Perinatal characteristics n (%)	
Outborn	22 (84.6)
Vaginal delivery	14 (53.8)
Age at presentation, Median (IQR, days)	24 (9–39)

Data are presented as mean ± standard deviation (SD), median (interquartile range [IQR]), or number (%), as appropriate. AGA, appropriate for gestational age; SGA, small for gestational age; LGA, large for gestational age.

### Risk factors and clinical characteristics

Exposure to broad-spectrum antibiotics, parenteral nutrition, central venous catheterization, and mechanical ventilation were commonly observed prior to the onset of fungal sepsis. Feeding difficulties and necrotizing enterocolitis were documented in a subset of neonates. The most common clinical presentations included respiratory distress, feed intolerance, and labile oxygen saturation suggestive of persistent pulmonary hypertension, along with other systemic manifestations. Clinical outcomes are also summarized in Table 2. Overall survival was 80.8% (five deaths, 19.2%); none of the non-survivors achieved culture clearance. During the study period, 352 preterm neonates were admitted, of whom 96 had sepsis of any cause; 22 of these 96 died of sepsis (sepsis-related mortality, 22.9%), and 27 preterm neonates died overall. Among the 26 neonates with fungal sepsis included in the study, 19 were preterm. These denominators are provided to contextualize the burden of fungal sepsis.

**Table 2:** Clinical Spectrum and outcomes of neonatal fungal sepsis.

Variable	Value
A. Prior Treatment Exposure	
A. Prior Treatment Exposure	
Prior antibiotic exposure, n (%)	23 (88.4)
Median duration of antibiotic exposure, days (IQR)	9 (5–15)
Duration of antibiotics ≥7 days, n (%)	17 (65.4)
	• Aminoglycosides 19 (73.1)
	• Carbapenems 14 (53.8)
	• Cephalosporins 11 (42.3)
	• Glycopeptides 4 (15.4)
Antibiotic classes used n (%)	• Fluoroquinolones 3 (11.5)
	• Polymyxins 3 (11.5)
	• Oxazolidinones 2 (7.7)
	• Penicillin 1 (3.8)
Antifungal prophylaxis, n (%)	5 (19.2)
Parenteral nutrition, n (%)	14 (53.8)
Duration of PN before sepsis, days (IQR)	9 (4–15)
Central venous catheter present, n (%)	9 (34.6)
Duration of catheterization before sepsis, days (IQR)	7 (4–10)
Mechanical ventilation before sepsis, n (%)	7 (26.9)
Duration of mechanical ventilation before sepsis, days (IQR)	7 (4–8)
Post-surgical fungal sepsis, n (%)	4 (15.4)
B. Enteral Feeding Characteristics	
Age at initiation of feeds, days (IQR)	12 (5–22)
Age at attainment of full feeds, days (IQR)	20 (9–30)
Feed intolerance, n (%)	16 (61.5)
Necrotizing enterocolitis* (any stage), n (%)	7 (26.9)
C. Clinical Manifestations at Presentation n (%)	
Respiratory/oxygenation	Respiratory distress 7 (26.9); labile oxygen saturation 5 (19.2); inability to wean from respiratory support 4 (15.4); apnea 3 (11.5); pulmonary haemorrhage 1 (3.8)
Gastrointestinal/feed-intolerance-related*	Feed intolerance 6 (23.1); abdominal distension 4 (15.4); loose stools 3 (11.5); bilious vomiting 1 (3.8); brown gastric aspirates 1 (3.8); bleeding per rectum 1 (3.8)
Metabolic/other	Hyperglycemia 3 (11.5); fever 3 (11.5); poor perfusion/delayed capillary refill 2 (7.7); hypoglycaemia 1 (3.8); lethargy 1 (3.8); seizure 1 (3.8); jaundice 1 (3.8)
D. Complications During Illness	
Neonatal cholestasis, n (%)	14 (53.8)
Acute kidney injury, n (%)	12 (46.1)
Inotropic support required, n (%)	5 (19.2)
E. Clinical Outcomes	
Duration of antifungal therapy, days (median, IQR)**	14 (7–54)
Length of hospital stay, days (median, IQR)	33 (12–112)
Survival, n (%)	21 (81)
Mortality, n (%)	5 (19)

Data are presented as mean ± standard deviation (SD), median (interquartile range [IQR]) or range, or number (%), as appropriate. Antibiotic classes are not mutually exclusive. PN, parenteral nutrition; NEC, necrotizing enterocolitis; AKI, acute kidney injury.

* Gastrointestinal/feeds and NEC related events occurred during episode of fungal sepsis.

** As per unit protocol antifungal therapy was continued for 14 days after the first documented sterile (culture-negative) blood culture: the reported value reflects total treatment duration.

### Laboratory and microbiological findings

Laboratory evaluation revealed frequent hematological abnormalities, including thrombocytopenia, neutropenia, and abnormal I:T ratio. Thrombocytopenia was present in 9 (34.6%), neutropenia in 5 (19.2%), and an abnormal I:T ratio in 11 (42.3%) neonates. The median haemoglobin was 11.4 g/dL (IQR 9.3–13.5), the median total leukocyte count was 12,150/mm^3^ (IQR 5,675–16,750), and the median platelet count was 148,500/mm^3^ (IQR 64,250–392,500). Inflammatory markers were markedly increased, and many neonates exhibited elevated serum creatinine and direct hyperbilirubinemia.

Microbiological evaluation revealed positive blood cultures in 24 (92.3%) neonates, with urine and cerebrospinal fluid cultures positive in 3 (11.5%) infants each. Blood isolates were predominantly Candida spp., with non-albicans Candida representing 16 (61.5%) and Candida albicans 5 (19.2%), while mixed and non-Candida fungi were less frequent. Antifungal susceptibility testing showed triazole sensitivity in 23 (88.5%) isolates, echinocandin sensitivity in 18 (69.2%), and polyene sensitivity in 17 (65.4%); 31.25% of non-albicans isolates were triazole-resistant. Fluconazole was the most commonly used antifungal, followed by voriconazole and amphotericin B, administered as monotherapy or in combination, with micafungin escalation in select cases.

### Radiological and imaging findings

**Figure 2:**
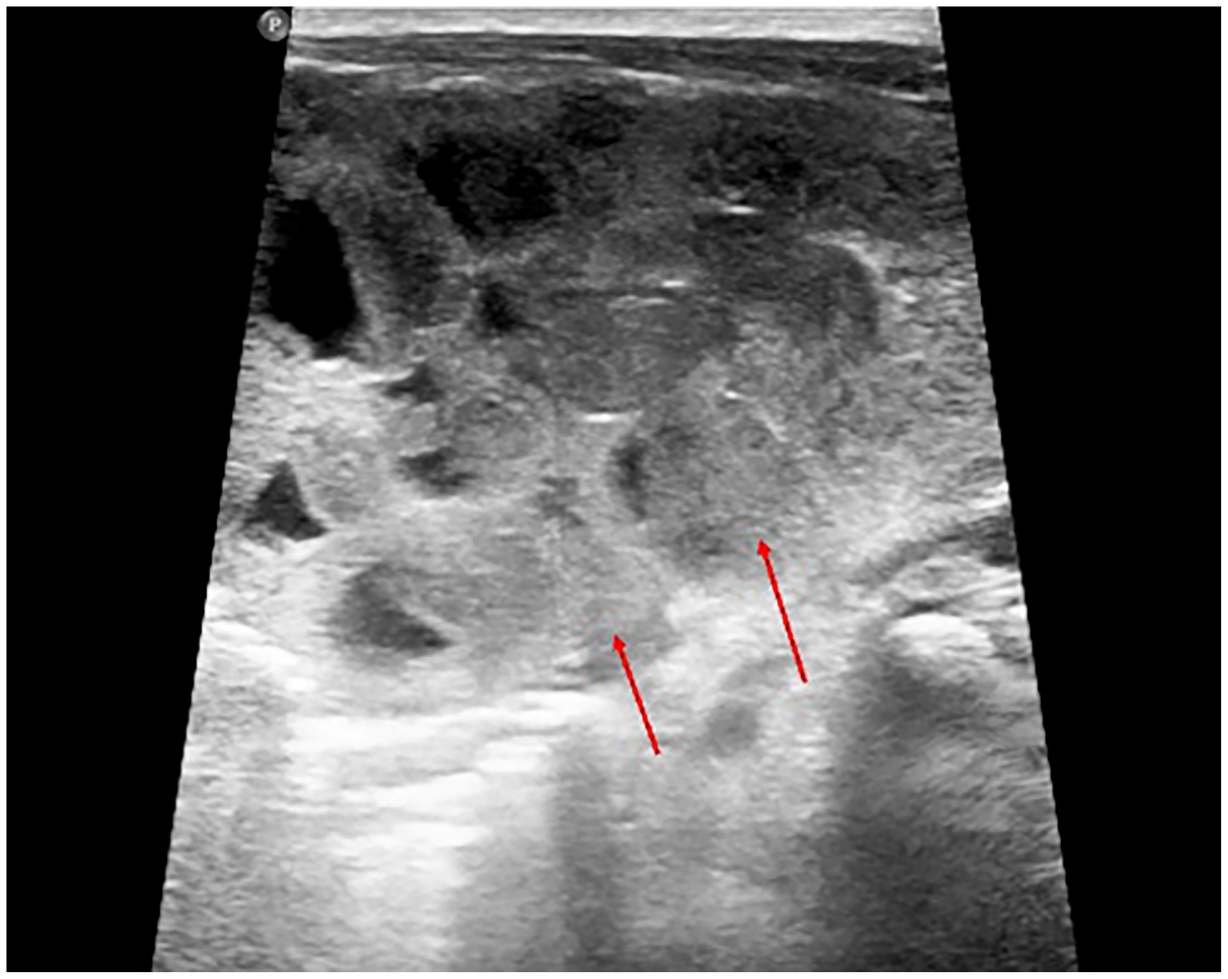
Ultrasonography in a late preterm, low-birth-weight male infant with culture-proven candidemia demonstrates bilateral renomegaly with increased renal echogenicity. Multiple well-defined hypoechoic lesions in the right renal parenchyma are consistent with renal microabscesses. Linear echogenic foci within both pelvicalyceal systems are suggestive of fungal balls. The arrows indicate the renal microabscesses.

Radiological investigations demonstrated abnormalities in multiple organ systems. Abdominal ultrasonography was abnormal in 6 (23.1%) neonates, with findings including hepatosplenomegaly with splenic microabscess (*n* = 1), bowel wall edema with ascites (*n* = 2), gallbladder sludge and wall thickening (*n* = 2), and reduced hepatic echogenicity (*n* = 1). Renal abnormalities were identified in 4 (15.4%) infants, including bilateral hydronephrosis with pyonephrosis (*n* = 1), echogenic foci suggestive of fungal balls (*n* = 1) (Figure 2), nephrocalcinosis with echogenic renal pyramids (*n* = 1), and pelvicalyceal hyperechoic lesions with hydronephrosis (*n* = 1).

**Figure 3:**
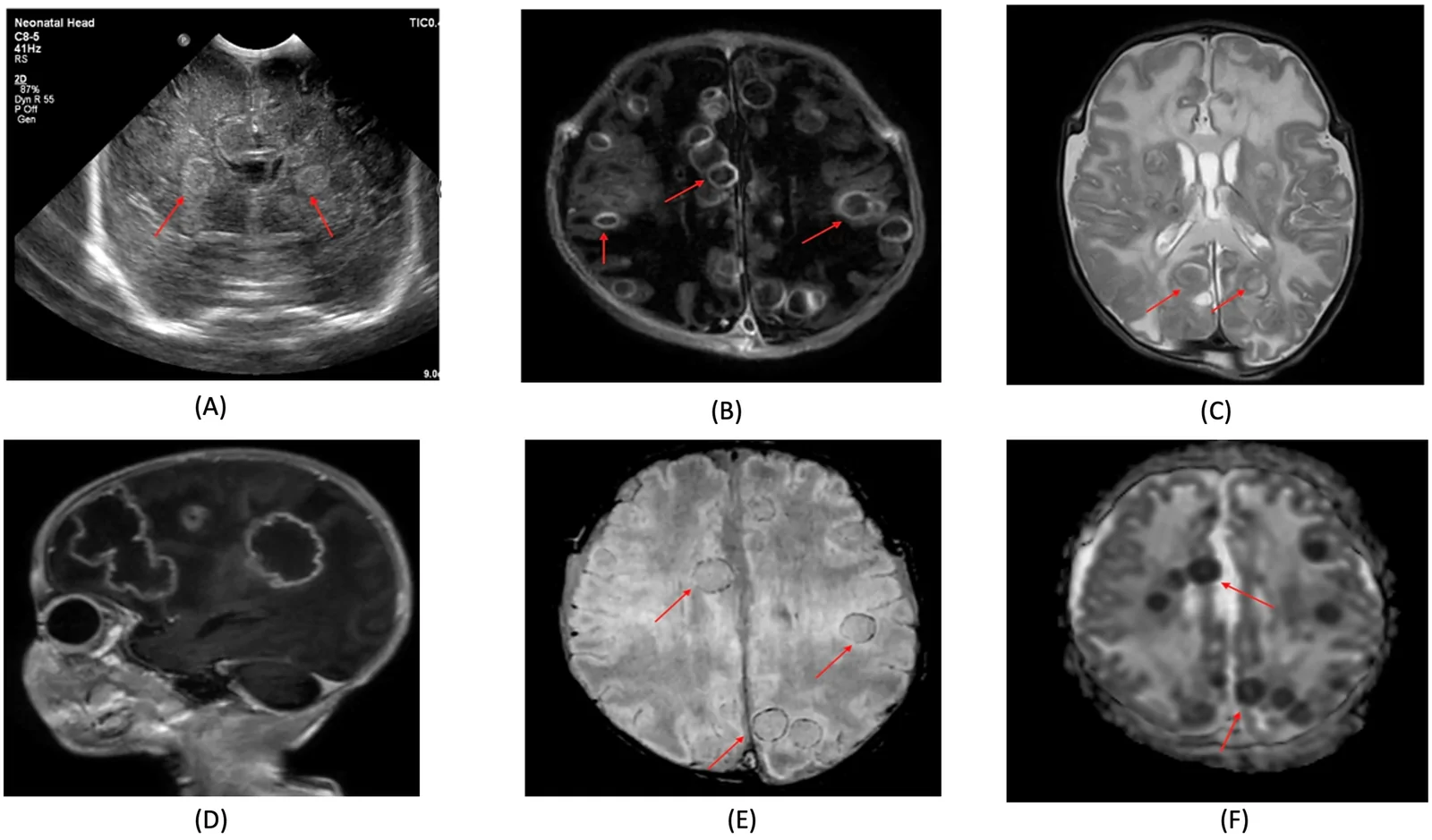
Neuroimaging findings in neonatal invasive candidiasis. **(A)** Cranial ultrasonography demonstrating bilateral hyperechoic lesions in the basal ganglia (arrows). **(B)** Axial post-contrast MRI demonstrating multiple ring-enhancing cerebral abscesses (arrows). **(C)** T2-weighted MRI showing focal parenchymal hyperintensities with surrounding edema (arrows). **(D)** Sagittal MRI depicting large cortical abscess with peripheral enhancement. **(E)** Susceptibility-weighted imaging demonstrating peripheral hemorrhagic areas (arrows). **(F)** Diffusion-weighted imaging demonstrating restricted diffusion within abscess cavities (arrows).

Brain ultrasonography demonstrated abnormalities in 3 (11.5%) neonates, including multiple cerebral abscesses in two infants and bilateral caudate hypoechogenicity in one infant (Figure 3A). Advanced imaging was performed in selected cases. Magnetic resonance imaging (MRI) of the brain demonstrated multiple well-defined abscesses in two neonates (Figures 3B–D, f) and area of peripheral hemorrhagic infarcts involving the frontal, temporal, and parietal lobes in one neonate (Figure 3E). T2-weighted imaging further demonstrated associated parenchymal edema (Figure 3C). MRI urography in one infant confirmed bilateral renal enlargement with pyonephrosis. Fundus examination performed in all neonates did not reveal evidence of endophthalmitis.

### Echocardiographic and hemodynamic findings

**Figure 4:**
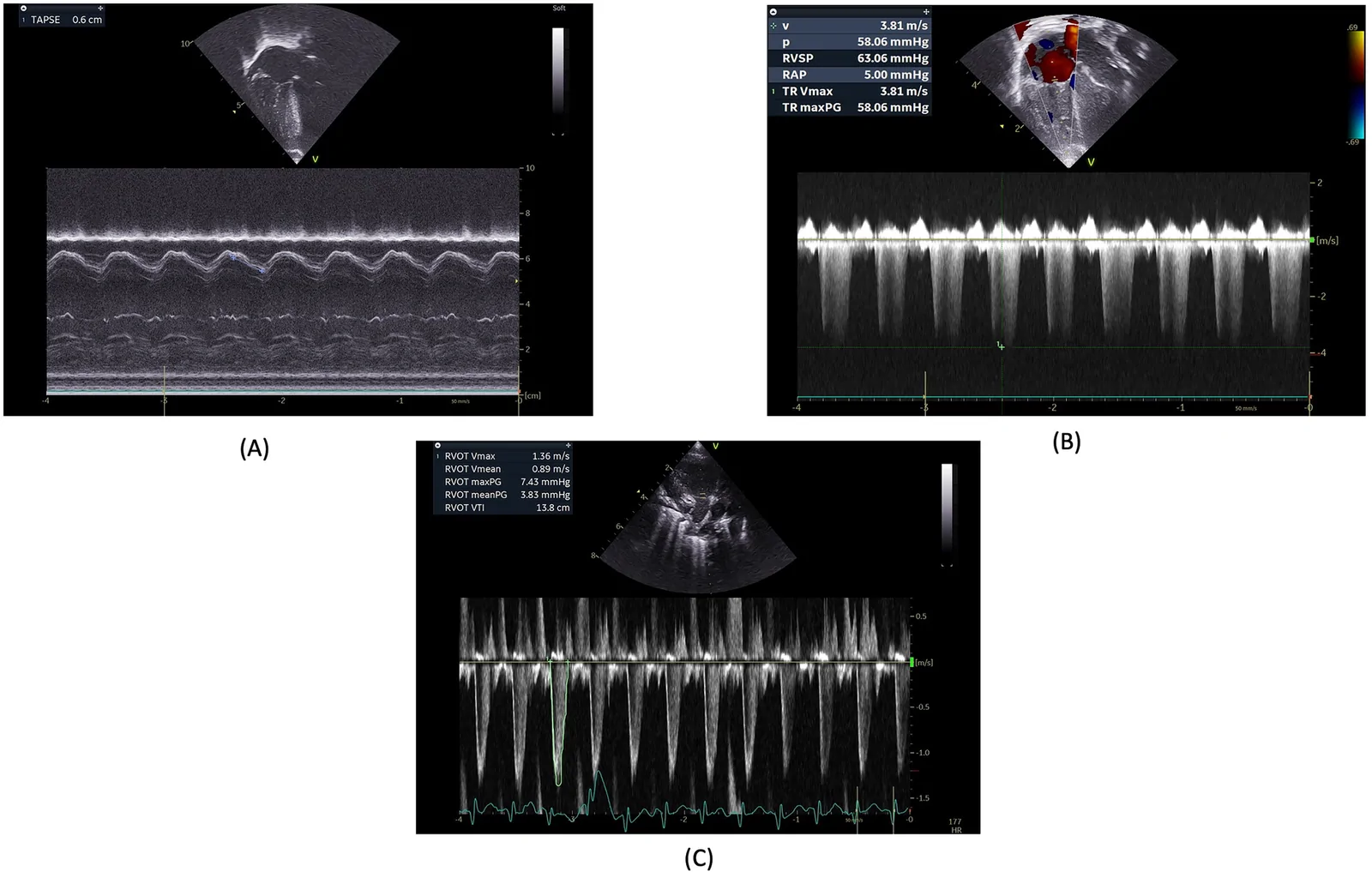
Representative functional echocardiographic findings in neonates with culture-proven fungal sepsis. The illustrated individual cases are representative examples of imaging findings and are not intended to reflect the overall cohort characteristics. **(a)** M-mode echocardiographic assessment of tricuspid annular plane systolic excursion (TAPSE) demonstrating reduced excursion of 0.6 cm (6 mm) in a term neonate with culture-proven fungal sepsis. **(b)** Continuous-wave Doppler across the tricuspid regurgitation (TR) jet demonstrating a peak velocity of 3.81 m/s, corresponding to a peak TR gradient of 58 mmHg and an estimated right ventricular systolic pressure (RVSP) of 63 mmHg (assuming right atrial pressure of 5 mmHg). **(c)** Pulse-wave of the right ventricular outflow tract (RVOT) demonstrating a velocity time integral (VTI) of 13.8 cm in a term neonate (birth weight 3.5 kg) with culture-proven fungal sepsis. The calculated right ventricular output (RVO) is approximately 346 mL/kg/min.

No valvular vegetations or echocardiographic evidence of infective endocarditis were identified in any neonate on dedicated screening by the pediatric cardiologist. Among survivors (*n* = 21), paired pre–post analysis of echocardiographic parameters was performed. The pre-treatment echocardiography was obtained at the time of clinical suspicion of sepsis, while the post-treatment echocardiography was performed after microbiological clearance (repeat culture negative). At baseline, neonates exhibited right ventricular dysfunction with features of reduced TAPSE (Figure 4A), elevated PASP (Figure 4B), and increased RVO (Figure 4C). Following treatment, there was a significant improvement in right ventricular systolic function, reflected by increased TAPSE (*p* < 0.05), along with a marked reduction in PASP and RVO (both *p* < 0.01). Diastolic dysfunction observed during the pre-treatment phase also showed significant reversal post-treatment, with improvement in both tricuspid and mitral E:A ratios (both *p* < 0.01). In contrast, left ventricular systolic function remained relatively preserved, with no statistically significant differences between pre- and post-treatment values for LVO, fractional shortening, and ejection fraction (all *p* > 0.05). Overall, the pre–post comparison highlights a predominantly right-sided ventricular dysfunction, pulmonary vascular, and biventricular diastolic derangement during active fungal sepsis, with significant reversibility following microbiological clearance. Changes in echocardiographic parameters before and after treatment are summarized in Table 3.

**Table 3:** Echocardiographic parameters before and after antifungal therapy in survivors.

**Parameter**	**Pre (mean ± SD)**	**Post (mean ± SD)**	***Δ*(mean change)**	***p*-value**
TAPSE (mm)	6.4 ± 1.2	7.8 ± 2.4	+1.4	<0.05
Tricuspid E:A	0.69 ± 0.1	0.90 ± 0.1	+0.2	<0.01
RVO (mL/kg/min)	315.0 ± 49.4	246.0 ± 27.1	−69.0	<0.01
PASP (mmHg)	28.5 ± 17.3	12.8 ± 4.2	−15.7	<0.01
Mitral E:A	0.70 ± 0.1	0.90 ± 0.1	+0.2	<0.01
LVO (mL/kg/min)	246.0 ± 68.0	226.0 ± 41.5	−20.0	0.26
FS (%)	27.8 ± 5.2	29.3 ± 6.2	+1.5	0.40
EF (%)	50.3 ± 4.9	52.0 ± 5.6	+1.7	0.30

Pre, at clinical suspicion of sepsis; post, after microbiological clearance (repeat culture negative). EF, ejection fraction; E:A, early-to-late ventricular filling velocity ratio; FS, fractional shortening; LVO, left ventricular output; PASP, pulmonary artery systolic pressure; RVO, right ventricular output; SD, standard deviation; TAPSE, tricuspid annular plane systolic excursion.

Differences in clinical and echocardiographic parameters were observed between survivors and non-survivors. All non-survivors required invasive ventilation with higher ventilatory requirements. They exhibited higher heart rates, lower systemic blood pressure, and a greater need for inotropic support during the acute phase of illness. Clinical findings were corroborated by echocardiography. Detailed hemodynamic comparisons are presented in Table 4; overall, echocardiography demonstrated features of hemodynamic compromise in non-survivors, consistent with the observed clinical severity. Among the five non-survivors, death was attributable to myocardial dysfunction. Sepsis and cardiovascular dysfunction were managed according to standard neonatal septic shock management guidelines and unit protocol, integrating clinical parameters and echocardiographic findings.

**Table 4:** Clinical variables and echocardiographic findings at baseline (time of clinical suspicion of fungal sepsis) in survivors and non-survivors of neonatal fungal sepsis.

**Parameter**	**Survivors (mean ± SD) (n = 21)**	**Non-survivors (mean ± SD) (*n* = 5)**
Heart rate (beats/min)	167.5 ± 9.6	173.8 ± 1.9
Systolic blood pressure (mmHg)	60.8 ± 8.5	48.4 ± 3.9
Diastolic blood pressure (mmHg)	44.8 ± 11.1	36.8 ± 4.4
Mean blood pressure (mmHg)	47.1 ± 2.6	36.4 ± 5.5
TAPSE (mm)	6.4 ± 1.2	4.8 ± 1.5
Tricuspid E:A	0.7 ± 0.1	0.6 ± 0.1
RVO (mL/kg/min)	315.0 ± 49.4	367.8 ± 48.2
PASP (mmHg)	28.5 ± 17.3	38.4 ± 23.6
Mitral E:A	0.7 ± 0.1	0.6 ± 0.1
LVO (mL/kg/min)	246.0 ± 68.0	221.5 ± 60.7
FS (%)	27.8 ± 5.2	25.3 ± 6.1
EF (%)	50.3 ± 4.9	48.6 ± 5.4

Owing to the small number of non-survivors (*n* = 5), groups are compared descriptively and no inferential *p*-values are reported. EF, ejection fraction; E:A, early-to-late ventricular filling velocity ratio; FS, fractional shortening; LVO, left ventricular output; PASP, pulmonary artery systolic pressure; RVO, right ventricular output; SD, standard deviation; TAPSE, tricuspid annular plane systolic excursion.

Clinical and echocardiographic variables were recorded at the time of the baseline echocardiogram, performed during the initial evaluation for suspected sepsis. Groups are compared descriptively owing to the small number of non-survivors.

## Discussion

In this retrospective cohort from a tertiary NICU in western India, invasive fungal sepsis constituted a notable proportion of culture-positive infections, accounting for 8.7% of cases, underscoring the persistent burden in resource-limited settings. This is broadly consistent with data from low- and middle-income countries, although direct comparisons are limited by differences in study populations. The NICHD Neonatal Research Network reported invasive candidiasis in 7% (320/4579) and 9.0% (137/1515) of ELBW infants (1, 2), while the NeoOBS cohort demonstrated substantial burden with 22% mortality across LMICs (3). Contemporary estimates report an incidence of 0.5%–2% overall, rising to 2%–20% in ELBW infants, with higher rates in developing countries (4). These findings suggest that the higher burden observed in our cohort may relate to cumulative NICU exposures and differences in infection prevention practices.

Neonatal invasive candidiasis predominantly affects preterm and low-birth-weight infants, though the degree of prematurity varies by setting. While NICHD data largely represent ELBW populations (1, 2), the NeoOBS cohort (median GA 30 weeks, birth weight 1,270 g) highlights involvement of relatively more mature neonates in LMICs (3). Our cohort mirrors this pattern, indicating that risk extends beyond ELBW infants in resource-limited settings. This has important clinical implications, as reliance solely on ELBW-based risk may delay recognition of fungal sepsis in larger preterm or even term neonates.

The risk factor profile in our study closely mirrors multicenter and review data, emphasizing cumulative NICU-related exposures. NICHD studies identified cephalosporin use, lack of enteral feeding, and low birth weight as independent risk factors, with central venous catheters, endotracheal tubes, and intravenous lipid administration as key modifiable contributors (1, 2). Contemporary data further highlight the role of antimicrobial pressure in increasing susceptibility to invasive candidiasis (4). Prolonged hospitalization and invasive device use further increase susceptibility, particularly in neonates with immature barrier and immune function (5). The clustering of antibiotic exposure, parenteral nutrition, and invasive support in our cohort is therefore consistent with established mechanisms of fungal overgrowth, mucosal translocation, and bloodstream invasion (4). Clinically, presentation in our cohort was non-specific, characterized by respiratory deterioration, feeding intolerance, and progressive hemodynamic instability. This aligns with existing reviews, where apnea, respiratory distress, feeding difficulty, and hemodynamic compromise are common and often indistinguishable from bacterial sepsis (6). Similar findings from Indian neonatal units report worsening respiratory distress, shock, necrotizing enterocolitis, thrombocytopenia, and frequent end-organ involvement in fungal infection (7).

A key microbiological finding was the predominance of non-albicans *Candida* species, consistent with evolving global epidemiology. In the NeoOBS cohort, *Candida albicans* accounted for 35%, *C. parapsilosis* 30%, and *C. auris* 14% of isolates, highlighting the substantial contribution of non-albicans species in LMICs (3). Contemporary reviews similarly report this shift across India, Asia, and Africa (4).

Consistent with this, antifungal susceptibility patterns in our cohort demonstrated azole resistance among non-albicans isolates. The NeoOBS study reported that while most Candida albicans isolates remained fluconazole susceptible, 59% of C. *parapsilosis* isolates were fluconazole resistant (3). Reviews further emphasize increasing resistance among non-albicans species, including C. *parapsilosis*, C. *krusei*, and C. *auris*, particularly in settings with widespread antimicrobial and antifungal exposure (4). These findings underscore the importance of routine species identification and susceptibility testing in neonatal fungal sepsis, particularly where empiric fluconazole use is common.

Another important microbiological consideration is the underestimation of invasive disease when diagnosis relies solely on blood cultures. In the NICHD 2006 cohort, among neonates with Candida meningitis, 48% had negative blood cultures, indicating that bloodstream isolation alone may significantly underestimate the burden of disseminated infection (1). This is highly relevant to our cohort, in which evidence of end-organ involvement was observed across multiple systems. Prior neonatal data have reported end-organ involvement in approximately 14% of neonates with candidemia, including renal abscesses, central nervous system infection, and less commonly ocular or cardiac disease (7). The pattern of renal and central nervous system involvement in our study is therefore consistent with the known hematogenous dissemination of Candida to highly vascular organs. Persistent candidemia and biofilm formation on intravascular devices likely contribute to this process by acting as reservoirs for ongoing organ seeding (4).

A distinctive and clinically important aspect of our study is the systematic characterization of hemodynamic alterations in neonatal fungal sepsis using functional echocardiography. During active infection, the cohort exhibited a consistent hemodynamic pattern marked by elevated right-sided cardiac output, increased PASP, and impaired diastolic filling, which normalized after microbiological clearance, while left ventricular systolic function remained comparatively preserved.

Sepsis-associated myocardial dysfunction is a well-recognized phenomenon, attributed to various inflammatory cytokines, myocardial depressant factors, and the complement cascade (9). Our observations demonstrating fungal sepsis induced myocardial suppression, were also observed in bacterial neonatal sepsis in previously published literature. Sumbaraju et al. found lower TAPSE in preterm septic neonates (10). In a prospective study of late-onset neonatal sepsis, Deshpande et al. reported elevated ventricular outputs in gram-negative sepsis (11). In a separate prospective study by the same authors, pulmonary hypertension was observed in 49% of septic neonates, findings similar to our study (10, 12).

In our study, neonates with fungal sepsis exhibited significantly deranged biventricular diastolic dysfunction, as characterized by the E:A ratio. This finding aligns only partially with existing literature regarding ventricular performance in bacterial sepsis, Awany et al., and Abdel-Hady reported no significant derangement in the tricuspid and mitral valve E:A ratio when comparing bacterial sepsis cases versus controls (13, 14). Conversely, Tomerak et al. and Sumbaraju et al. identified a marked decrease in the mitral E:A ratio in septic neonates (10, 15). However, Sumbaraju et al. did not find a significantly abnormal tricuspid E:A ratio between two groups (10).

An apparent paradox in our findings is the coexistence of reduced TAPSE with an elevated right ventricular output during active infection. These indices capture different aspects of right ventricular performance: TAPSE reflects longitudinal systolic excursion of the right ventricular free wall and is sensitive to both intrinsic contractility and loading conditions, whereas RVO is a global flow measure determined by stroke volume and heart rate. In the hyperdynamic, vasodilated circulatory state that characterizes early sepsis, reduced systemic vascular resistance, tachycardia, and increased venous return can sustain or even increase global output despite impaired longitudinal contractility. A concurrently elevated PASP indicates increased right ventricular afterload, which can further depress longitudinal shortening (and thus TAPSE) while output is transiently maintained through these compensatory mechanisms. The subsequent normalization of TAPSE, PASP, and RVO after microbiological clearance is consistent with a reversible, load-dependent process rather than fixed structural dysfunction. In the absence of invasive or advanced strain-based measurements, these mechanistic interpretations should be regarded as hypothesis-generating (9).

To place these observations in context, we compared our findings with previously published hemodynamic data in neonatal bacterial sepsis (Supplementary Table S1). Several features overlap across etiologies: reduced TAPSE, elevated pulmonary pressures, increased ventricular output, and diastolic impairment have each been reported in bacterial sepsis cohorts (10–12, 15). Other findings are less consistent; for example, tricuspid and mitral E:A derangement was not observed in some bacterial sepsis studies (13, 14). Because our cohort lacked a contemporaneous bacterial sepsis comparator, these similarities and differences must be interpreted cautiously, and the observed pattern may substantially reflect a shared pathophysiological response to severe neonatal infection rather than a fungal-specific signature. It cannot be concluded that the hemodynamic profile described here is unique to fungal sepsis. Prospective, adequately powered studies that directly compare fungal and bacterial sepsis using standardized echocardiographic protocols are required to determine whether etiology-specific hemodynamic differences truly exist.

Our findings in this fungal sepsis cohort parallel the physiologic patterns described in bacterial sepsis, suggesting a reversible pattern of hyperdynamic circulation, pulmonary vascular involvement, and diastolic dysfunction that improves following infection clearance, with primarily right ventricular involvement, thereby supporting the role of functional echocardiography in this population. A recent systematic review of functional echocardiography in septic newborns (12 studies, 438 septic neonates) similarly reported an increased risk of pulmonary hypertension and left ventricular diastolic dysfunction together with a warm shock physiology characterized by elevated cardiac outputs, and concluded that wider use of neonatologist-performed echocardiography is needed to guide hemodynamics-based management (16). Future prospective research with a bacterial sepsis comparator is required to explore ventricular dysfunction in neonatal fungal sepsis.

A key strength of this study is the systematic and longitudinal assessment of hemodynamic alterations in neonatal fungal sepsis using functional echocardiography, performed during active infection and after microbiological clearance, allowing dynamic evaluation of cardiovascular changes. To our knowledge, this is among the first studies to specifically characterize the hemodynamic profile of neonatal fungal sepsis, thereby addressing an important gap in the literature. In addition, the integration of clinical, microbiological, and echocardiographic data from a real-world tertiary neonatal intensive care unit in a resource-limited setting enhances the clinical relevance and applicability of our findings to similar contexts.

However, several limitations should be considered. The retrospective single-center design precludes causal inference and is subject to selection and information bias. The relatively small sample size limits statistical power, particularly for subgroup analyses. The absence of a bacterial sepsis comparator precludes determining whether the observed hemodynamic changes are specific to fungal sepsis or represent a common response to neonatal sepsis. Intra- and inter-observer reproducibility of echocardiographic measurements was not assessed, measurements were not blinded to clinical status, and no adjustment for multiple comparisons was performed, potentially introducing measurement bias and increasing the risk of type I error. As a single-center study from western India, the findings may not be generalizable to other settings or populations. Finally, long-term neurodevelopmental outcomes were not assessed.

## Conclusion

Neonatal fungal sepsis remains an important cause of late-onset infection in NICUs, particularly among preterm and low-birth-weight infants. Our study highlights the predominance of non-albicans Candida, healthcare-associated risk factors, and frequent multi-organ involvement. Though our study did not include a bacterial sepsis comparator cohort, published literature suggests that the observed hemodynamic alterations are broadly consistent with those reported in neonatal bacterial sepsis. Functional echocardiography demonstrated reversible myocardial dysfunction, predominantly affecting right ventricular function following resolution of sepsis. Early diagnosis, timely initiation of appropriate antifungal therapy, and rigorous infection prevention practices remain essential to improve neonatal outcomes.

Future prospective multicentric studies incorporating a bacterial sepsis comparator cohort and standardized functional echocardiographic assessment are warranted to identify pathogen-specific hemodynamic differences and improve the understanding of cardiovascular involvement in neonatal sepsis.

## Data Availability

The original contributions presented in the study are included in the article/Supplementary Material, further inquiries can be directed to the corresponding author.
