# Supplementary material for: Hemodynamic profile of neonatal fungal sepsis with insights into the disease spectrum: a retrospective study
Source: Front Pediatr. 2026 Aug 21;14:1905411. doi: 10.3389/fped.2026.1905411 (PMC13541655; doi:10.3389/fped.2026.1905411)
Supplement: Supplementary file 1 [file Table1.docx]

**Supplementary Table S1.**

**Comparison of Published Hemodynamic Findings in Neonatal Bacterial Sepsis with the Objective Hemodynamic Findings of the Present Fungal Sepsis Cohort**

| **Study / Year** | **Population & design** | **Variable(s) evaluated** | **Reported finding in neonatal bacterial sepsis (published literature)** | **Corresponding measured value in present fungal cohort (pre → post; p)** | **Concordance vs present cohort** | **Clinical relevance** |
| --- | --- | --- | --- | --- | --- | --- |
| Sumbaraju et al.^1^ (2024) | Preterm neonates with sepsis; observational, cases vs controls (further design specifics: Not reported) | TAPSE; myocardial performance (Tei) index; tricuspid and mitral E:A | Lower TAPSE in preterm septic neonates and reduced mitral E:A; no significant difference in tricuspid E:A between groups (numeric values: Not reported here) | **TAPSE 6.4 → 7.8 mm (p<0.05); tricuspid E:A 0.69 → 0.90 and mitral E:A 0.70 → 0.90 (both p<0.01)** | Concordant for reduced TAPSE and reduced mitral E:A; we additionally found reduced tricuspid E:A | Supports echocardiographic detection of RV systolic and diastolic dysfunction in neonatal sepsis |
| Deshpande et al.^2^  (2017) | Neonates with late-onset sepsis (gram-negative subgroup); prospective observational | LVO and RVO (cardiac output) | Elevated ventricular outputs in gram-negative late-onset sepsis (numeric values: Not reported here) | **RVO 315.0 → 246.0 mL/kg/min (p<0.01); LVO 246.0 → 226.0 mL/kg/min (p=0.26, NS)** | Concordant: high-output, hyperdynamic RV state that fell after clearance | High-output physiology in bacterial LOS parallels the fungal cohort |
| Deshpande et al.^3^  (2021) | Neonates with late-onset sepsis; prospective, functional echocardiography | PASP; prevalence of pulmonary hypertension | Pulmonary hypertension present in 49% of septic neonates | **PASP 28.5 → 12.8 mmHg (p<0.01)** | Concordant: elevated PASP with pulmonary vascular involvement, reversible after clearance | Pulmonary vascular involvement is common in neonatal sepsis irrespective of organism |
| Awany (Awny) et al.^4^  (2016) | Neonates with sepsis vs controls; comparative (design specifics: Not reported) | Tissue-Doppler and speckle-tracking indices; tricuspid and mitral E:A | No significant tricuspid or mitral E:A derangement between cases and controls; myocardial dysfunction detectable by tissue Doppler/strain (numeric values: Not reported here) | **Tricuspid E:A 0.69 → 0.90; mitral E:A 0.70 → 0.90 (both p<0.01)** | Partly discordant: myocardial involvement shared, but we found significant biventricular E:A reduction | Diastolic (E:A) changes in neonatal sepsis are inconsistent across cohorts |
| Abdel-Hady et al.^5^ (2012) | Neonates with sepsis vs controls; comparative tissue-Doppler study (design specifics: Not reported) | Tissue-Doppler indices; myocardial function; E:A | Myocardial dysfunction by tissue Doppler; no significant tricuspid/mitral E:A derangement reported (numeric values: Not reported here) | **Tricuspid E:A 0.69 → 0.90; mitral E:A 0.70 → 0.90 (both p<0.01); LV systolic preserved (FS 27.8 → 29.3%, p=0.40; EF 50.3 → 52.0%, p=0.30)** | Concordant for myocardial involvement with preserved LV systolic function; discordant for E:A derangement | Tissue Doppler can detect sepsis-associated myocardial dysfunction |
| Tomerak et al.^6^  (2012) | Septic neonates; echocardiography performed early in sepsis (design specifics: Not reported) | Conventional echocardiographic indices, including mitral E:A | Marked decrease in mitral E:A in septic neonates (numeric values: Not reported here) | **Mitral E:A 0.70 → 0.90 (p<0.01)** | Concordant: reduced mitral E:A during acute sepsis, reversible after clearance | Early echocardiography adds diastolic functional information in neonatal sepsis |
| Duignan et al.^7^  (2024) | Neonatal sepsis (general); narrative review of mechanisms and pathophysiology | Mechanistic — inflammatory cytokines, myocardial depressant factors, complement cascade | Sepsis-associated myocardial dysfunction driven by inflammatory mediators; typically reversible (no primary hemodynamic dataset) | **Reversible pattern: all deranged RV indices (TAPSE, PASP, RVO, tricuspid/mitral E:A) normalized after microbiological clearance; LV systolic function preserved throughout** | Concordant: our reversible, load-dependent pattern fits the proposed mechanism | Framework for interpreting reversible sepsis-associated myocardial dysfunction |
| ***Values for the present fungal sepsis cohort are shown as pre-treatment → post-microbiological clearance in survivors (n = 21), with paired p values. Published bacterial sepsis findings summarize the changes reported in the respective studies. “Not reported” indicates that the original study did not provide the corresponding numerical value. Abbreviations:*** *E:A, early-to-late ventricular filling velocity ratio; EF, ejection fraction; FS, fractional shortening; LOS, late-onset sepsis; LV, left ventricle; LVO, left ventricular output; NS, not significant; PASP, pulmonary artery systolic pressure; RV, right ventricle; RVO, right ventricular output; TAPSE, tricuspid annular plane systolic excursion.* | | | | | | |

References:

1. Sumbaraju SL, Nayak K, Prabhu S, Nayak V, Prabhu KP, Lewis LE. Myocardial performance imaging for the early identification of cardiac dysfunction in neonates with sepsis. Int J Cardiovasc Imaging. 2024 Jul;40(7):1435-1444. doi: 10.1007/s10554-024-03120-z. Epub 2024 Jun 22. PMID: 38907854; PMCID: PMC11258056.
2. Deshpande S, Suryawanshi P, Chaudhary N, Maheshwari R. Cardiac output in late onset neonatal sepsis. J Clin Diagn Res. 2017;11(11):SC25-SC28. doi: 10.7860/JCDR/2017/30312.10871
3. Deshpande S, Suryawanshi P, Holkar S, Singh Y, Yengkhom R, Klimek J, Gupta S. Pulmonary hypertension in late onset neonatal sepsis using functional echocardiography: a prospective study. J Ultrasound. 2022 Jun;25(2):233-239. doi: 10.1007/s40477-021-00590-y. Epub 2021 May 15. PMID: 33991307; PMCID: PMC9148354.
4. Awany M, Tolba O, Al-Biltagi M, Al-Asy H, El-Mahdy H. Cardiac functions by tissue Doppler and Speckle tracking echocardiography in neonatal sepsis and its correlation with sepsis markers and cardiac troponin-T. J Ped Neonatal Care. 2016;5(3):184-. DOI:10.15406/jpnc.2016.05.00184
5. Abdel-Hady HE, Matter MK, El-Arman MM. Myocardial dysfunction in neonatal sepsis: a tissue Doppler imaging study. Pediatr Crit Care Med. 2012 May;13(3):318-23. doi: 10.1097/PCC.0b013e3182257b6b. PMID: 21725277.
6. Tomerak RH, El-Badawy AA, Hussein G, Kamel NR, Razak AR. Echocardiogram done early in neonatal sepsis: what does it add? J Investig Med. 2012 Apr;60(4):680-4. doi: 10.2310/JIM.0b013e318249fc95. PMID: 22373661.
7. Duignan SM, Lakshminrusimha S, Armstrong K, de Boode WP, El-Khuffash A, Franklin O, Molloy EJ; Infection, Inflammation, Immunology and Immunisation (I4) section of the European Society for Paediatric Research (ESPR). Neonatal sepsis and cardiovascular dysfunction I: mechanisms and pathophysiology. Pediatr Res. 2024 Apr;95(5):1207-1216. doi: 10.1038/s41390-023-02926-2. Epub 2023 Dec 4. PMID: 38044334.
